# Nanoparticles advanced from preclinical studies to clinical trials for lung cancer therapy

**DOI:** 10.1186/s12645-023-00174-x

**Published:** 2023-03-28

**Authors:** Yifan Liu, Wenxu Cheng, HongYi Xin, Ran Liu, Qinqi Wang, Wenqi Cai, Xiaochun Peng, Fuyuan Yang, HongWu Xin

**Affiliations:** 1 Laboratory of Oncology, Center for Molecular Medicine, School of Basic Medicine, Health Science Center, Yangtze University, 1 Nanhuan Road, Jingzhou, 434023 Hubei China; 2 Jingzhou Hospital Affiliated to Yangtze University, Yangtze University, Jingzhou, 434023 Hubei China; 3 The Doctoral Scientific Research Center, People’s Hospital of Lianjiang, Guangdong, 524400 China; 4 The Doctoral Scientific Research Center, People’s Hospital of Lianjiang, Affiliated to Guangdong Medical University, Guangdong, 524400 China; 5 Department of Pathophysiology, School of Basic Medicine, Health Science Center, Yangtze University, Jingzhou, 434023 Hubei China; 6 Xinzhou Traditional Chinese Medicine Hospital, Zhongnan Hospital of Wuhan University (Xinzhou), Hubei, 430000 China; 7 Department of Physiology, School of Basic Medicine, Health Science Center, Yangtze University, 1 Nanhuan Road, Jingzhou, 434023 Hubei China; 8 Department of Biochemistry and Molecular Biology, School of Basic Medicine, Health Science Center, Yangtze University, Jingzhou, 434023 Hubei China; 9 Research Center of Molecular Medicine, Medical College of Chifeng University, Inner Mongolian Autonomous Region, Chifeng, 024000 China

**Keywords:** Lung cancer, Nanoparticle, Immune targeted therapy, Nanotherapy

## Abstract

Lung cancer is the leading cause of cancer mortality. As a heterogeneous disease, it has different subtypes and various treatment modalities. In addition to conventional surgery, radiotherapy and chemotherapy, targeted therapy and immunotherapy have also been applied in the clinics. However, drug resistance and systemic toxicity still cannot be avoided. Based on the unique properties of nanoparticles, it provides a new idea for lung cancer therapy, especially for targeted immunotherapy. When nanoparticles are used as carriers of drugs with special physical properties, the nanodrug delivery system ensures the accuracy of targeting and the stability of drugs while increasing the permeability and the aggregation of drugs in tumor tissues, showing good anti-tumor effects. This review introduces the properties of various nanoparticles including polymer nanoparticles, liposome nanoparticles, quantum dots, dendrimers, and gold nanoparticles and their applications in tumor tissues. In addition, the specific application of nanoparticle-based drug delivery for lung cancer therapy in preclinical studies and clinical trials is discussed.

## Introduction

Lung cancer is a malignant disease that is a public problem facing the world and is growing at an alarming rate every year (Liu et al. 2023). Globally, there are an estimated 2 million new cases and 1.76 million deaths per year (Thai et al. 2021). When it comes to lung cancer, we usually think it is closely related to smoking. From the data of the studies, we know that without intervention in smoking, the risk of lung cancer in boys will be 9.6%., and the risk of lung cancer in girls would be 4.3% (Villeneuve and Mao 1994). However, lung cancer is much more than smoking, it is also associated with differences in geography, ethnicity, gender, socioeconomic and social class. In addition, environmental factors such as industrial toxins and exhaust pollution are also risk factors for the development of lung cancer (de Sousa and Carvalho 2018; Raman et al. 2022). It is evident that it is extremely urgent to overcome the challenge of lung cancer treatment technology. Like most tumors, lung cancer consists of molecular subtypes and clonal stages with different characteristics and is a heterogeneous disease (de Sousa and Carvalho 2018), which is the reason why lung cancer cannot be well-controlled with a single therapy. Usually, the treatment cycle of lung cancer is long and complex and may involve multiple therapeutic approaches, such as surgery, radiotherapy, chemotherapy, immunotherapy, targeted drugs, etc. (Vinod and Hau 2020). In recent years, the emergence of nanotechnology has greatly improved the therapeutic effect of different treatment modalities for lung cancer patients. Many studies have shown that nanoparticle-based drug delivery systems provide a promising platform for the targeted delivery of traditional chemotherapy drugs and new small molecule therapeutic agents (Doroudian et al. 2021). This is because the traditional delivery mode of immune drugs to tumor cells is oral or intravenous injection, which leads to the spread of drugs throughout the body. More or less, there will be a low concentration of drugs in tumor cells and the accumulation of drugs in normal tissues to destroy normal tissues. The enhanced permeability and retention effect of nanoparticles make nanoparticles accumulate more in tumor tissues, so nanomedicine opens up more possibilities for drug delivery (Suzuki and Cheung 2015). Therefore, with the progress of medical technology and medical level, doctors are no longer obsessed with cytotoxic treatment methods, but adopt personalized treatment plans (Herbst et al. 2018). The application of nanoparticles not only improves the therapeutic index of lung cancer but also reduces the drug dose required. Nanodrug delivery systems also offer the advantages of precise control of drug release, thereby reducing toxic side effects, controlling biodistribution and accelerating effects or reactions.

## Background of lung cancer and treatment modalities

### Background

According to the traditional classification of lung cancer, it can be divided into non-small cell carcinoma (NSCLC) and small cell carcinoma (SCLC), of which NSCLC accounts for 85%, SCLC accounts for 15% (Zhang et al. 2018). In clinical practice, chemotherapy is the mainstay of first-line treatment for metastatic small cell carcinoma, using a combination of Etoposide or Irinotecan with platinum, and chest radiation therapy should be promptly administered to patients without metastases. Unfortunately, few second-line agents are approved, and although Topotecan is the standard second-line option, it is also not available for all patients due to its mediocre efficacy and severe hematologic toxicity (Yang et al. 2019). Over the years, some progress has been made in immunotherapy for SCLC, and a review by Sen Yang et al. mentioned that Nivolumab, Pembrolizumab, Atezolizumab, and Durvalumab significantly improved the outcome of SCLC. Therefore, the future outlook for SCLC lies in new approaches to the integration of immunotherapy and small molecule TKI (Tyrosine kinase inhibitor) drugs (Yang et al. 2019). Common subtypes of NSCLC include large cell carcinoma, lung squamous carcinoma (LUSC) and lung adenocarcinoma (LUAD). LUAD is a rare and aggressive tumor that occurs in the lung and gastrointestinal tract in most cases, and platinum-based therapy combined with Etoposide or Irinotecan remains the first-line treatment option (Masso-Valles et al. 2020). Although patients with LUSC represent a minority in clinical practice and are rarely talked about in targeted therapies, because LUSC does not produce frequent drug target mutations and has a more durable immune response, LUAD predominates in lung cancer and most lung cancer patients are classified as lung adenocarcinoma (Herbst et al. 2018; Imyanitov et al. 2021). For patients with NSCLS, if N2 lymph node involvement can be detected during surgery, we can define them as early stage, which can be classified as stage I, II and IIIA, and surgical radical treatment is usually recommended in this case (Alexander et al. 2020). For stage III patients, who are candidates for non-surgical patients, the current standard is concurrent radiotherapy followed by immunotherapy (Alexander et al. 2020). In clinical practice, for stage II and IIIA patients, adjuvant platinum-based chemotherapy is recommended, which has been shown to reduce mortality but has a relatively high rate of eventual recurrence and cytotoxicity (Pignon et al. 2008). Therefore, a multifaceted and multidisciplinary reflection and discussion should be carried out before treatment to achieve the best therapeutic outcome. In recent years, targeted therapies have gradually come into the public eye, and their combination with immunotherapy has led to significant improvements in the treatment of some patients with advanced NSCLC (Herbst et al. 2018).

### Targeted therapy

There are many proteins on the surface of lung cancer cells, such as the EGFR family, ALK, ROS1, MET, RET, NTRK, etc., and when they are mutated, they are the best targeting sites. Therefore, a series of rapidly developing drugs are directed against them (Table 1). The epidermal growth factor receptor (EGFR) is a membrane receptor from the tyrosine kinase group (Rodak et al. 2021), which is a key regulator of cell proliferation, differentiation, division, survival, and cancer development (Qin et al. 2019). Therefore, it is recognized as the most potent target site (Dong et al. 2021). When mutated, EGFR promotes the generation of blood vessels and lymphatic vessels in tumor cells and inhibits apoptotic pathways and sustained proliferation (Zhang et al. 2020). To treat lung cancer due to EGFR mutations, epidermal growth factor receptor–tyrosine kinase inhibitor (EGFR–TKI) immune-targeted therapy plays an important role, and the first-generation ATP-competitive reversible inhibitors including Gefitinib, Erlotinib and Icotinib are currently the first-line treatment for patients with advanced EGFR mutations (Ye et al. 2021). For the first generation inhibitors the biggest problem is the mutation of T790M, which increases the ATP affinity of the receptor and spatially prevents drug binding, leading to the development of drug resistance. The second-generation EGFR–TKIS irreversibly binds competitively to the ATP binding site, which was created to address the basis of drug resistance of one generation of drugs, and it includes Afatinib and Dacmitinib, which overcome the resistance caused by the T790MD mutation, but it is ineffective, due to the reduced selectivity of the drug for EGFR mutants and the increased dose that leads to toxicity (Harrison et al. 2020). The third-generation drug is highly selective, namely, Osimertinib, which not only overcomes the problems caused by T790M mutations, but also targets EGFR mutations and T790M mutations (Ye et al. 2021).The FLAURA clinical trial also demonstrated the superiority of Osimertinib (Rodak et al. 2021). However, third-generation TKI also has some drawbacks, as it has shown serious toxicity during treatment and increased toxic reactions when used in combination with immunologic agents, as evidenced in patients who have previously received immunotherapy (Kauffmann-Guerrero et al. 2021). Mesenchymal lymphoma kinase (ALK) is involved in a wide range of cancers, from very rare cancers to ubiquitous NSCLC, and ALK activation in cancer is caused by overexpression and mutation of full-length ALK4–6, which seems to play a particularly important role in tumor drive when ALK translocations result in fusion with another gene (Hallberg and Palmer 2013). Several ALK TKI have been used in clinical treatment. After ALK was recognized as a target for NSCLC in 2007, the first generation drug Crizotinib was used in clinical studies in NSCLC in 2013 (Solomon et al. 2014). The emergence of drug resistance and tumor recurrence after 9 months, along with liver failure, led to the accelerated approval of second-generation TKI by the FDA, which included Ceritinib, Alectinib, and Brigatinib; however, resistance eventually developed due to mutations in ALK, while the third-generation TKI drug Lorlatinib had a mutation-inhibiting ability. In a clinical study, it demonstrated a very high therapeutic effect (Rodak et al. 2021; Solomon et al. 2018). At the same time, it has also shown common side effects, with some patients developing hypercholesterolemia and hypertriglyceridemia, and some having to stop treatment because of side effects (Shaw et al. 2019). ROS1 is closely related to cell differentiation and growth, and when it undergoes rearrangement it is suggested to be associated with cancer development (Rimkunas et al. 2012). Since ROS1 has a similar structure to ALK, Crizotinib was also used in cancers caused by ROS1 mutations, but of course resistance inevitably emerged. The poor intracranial permeability of Crizotinib led to the development of intracranial metastases in a proportion of NSCLC patients in advanced stages, which was a major cause of death (Patil et al. 2018). Then, Entretinib and Lorlatinib were clinically used, although it also has low general efficacy and high side effects including neurological and cardiovascular disease (Rodak et al. 2021). MET plays a driving role in a proportion of patients with lung cancer development (Mitiushkina et al. 2019). Clinical inhibitors targeting MET mutation, such as Capmatinib, Tepotinib, have some therapeutic effect on MET-induced NSCLC but unfortunately do not have a significant response rate. RET protein is a membrane tyrosine kinase receptor whose translocation leads to NSCLC. The drugs targeting RET protein clinically include Cabozantinib, Vandetanib and Sunitinib, which also face some problems, such as low survival. Mutations in the NTRK1 (Neurotrophic Receptor Tyrosine Kinase 1) gene are also involved in tumor transformation, and the immune-targeted drugs such as entrectinib and Larotrectinib are clinically used (Imyanitov et al. 2021; Rodak et al. 2021).

**Table 1:** Studies on targeted therapy for lung cancer

Target	Effects on the organism	Drugs	Limitations	References
EGFR	A key factor in cell proliferation, differentiation, division, survival, and cancer development	I: Gefitinib,Erlotinib,Icotinib	Produce limitations, and the third-generation drug Osimertinib has severe toxicity	(Ye et al. 2021)
		II: Afatinib,Dacomitinib		
		III: Osimertinib		
ALK	Control of cell cycle cell growth cell differentiation and anti-apoptosis	I: Crizotinib	Develops drug resistance, relapses after 9 months, and leads to liver failure	(Rodak et al. 2021)
		II: Ceritinib, Alectinib, Brigatinib	ALK point mutations lead to drug resistance	
		III: Lorlatinib	Significant side effects, 80% produce hypercholesterolemia	
ROS1	Regulation of cell growth and differentiation	Crizotinib Entretinib, lorlatinib	Low efficacy and high side effects, including neurological and cardiovascular disease	(Patil et al. 2018)
MET	MET proliferation, mutation, and fusion are associated with related tumorigenesis	Capmatinib, Tepotinib	Low response to treatment and mutations leading to drug resistance	(Mitiushkina et al. 2019)
RET	Mutations cause NSCLC to develop	Cabozantinib, vandetanib, sunitinib	Low response rate and low survival	(Rodak et al. 2021)
NTRK1	Regulation of cell growth and differentiation	Entretinib larotrectinib	Low response rate, no data on drug resistance yet	(Imyanitov et al. 2021)

Through the above introduction, we can understand that no matter what type of lung cancer is seriously harmful to human life and health, different types of lung cancer treatment methods are not the same, but they are all developing towards the trend of combined treatment and individualized treatment. From the summary of the treatment modalities available so far, it is evident that immunotherapy, when combined with targeted therapy, solves many clinical problems and greatly improves the survival rate of the disease. However, there are still many problems that need to be solved, such as the maximum efficacy of drugs due to poor permeability, the toxicity of drugs that damage other normal tissues except tumor cells, and the high metabolism of cancer cells, which reduce the efficacy of drugs. To solve these problems, it is necessary to start from the drug delivery system, and to have a deep understanding of the tumor immune tumor microenvironment (Wang et al. 2020). These urgent problems need to be solved due to the emergence of nanotechnology, which brings new benefits to patients with lung cancer. If immune targeted therapy is used based on the advantages of nanoparticles, it will achieve more superior therapeutic effects.

## Properties of nanoparticles and their applications in tumors

With the development of nanotechnology and medical technology, nanoparticles can be composed of different components, Below we will introduce several commonly used nanoparticles (Table 2).

**Table 2:** Diverse types of nanoparticles

Type	Discovery Time	Size (nm)	Advantage	First delivery	First delivery time	References
Polymeric nanoparticles	Unknown	1–1000	Good drug stability;	Abraxane	2005	(Aghebati-Maleki et al. 2020)
			Good endothelial permeability;			
			Accurate localization at tumor tissues			
Liposomes	1961	Varies with composition	Protection from degradation;	Doxil	1995	(Almeida et al. 2020)
			Reducing drug toxicity;			
			Biocompatibility;			
			Proper targeting			
Gold nanoparticles	1857	3–200	Optical and electronic properties;	TNF	2004	(Paciotti et al. 2004)
			Stable			
Quantum dots	1980	4–12	Small size;	Captopril	2006	(Wang and Chen 2011)
			Light stable and fluorescent			
Dendrimers	1985	2–10	High drug loading capacity;	Vivagel	2007	(Kim et al. 2018)
			Surface modifications possible;			
			Correctly targeting specific tissues or cells			
Carbon Nanotubes	1976	0.4–100	Stability and flexibility;	EPO	2005	(Saito et al. 2009)
			High ability to penetrate cell membranes;			
			High drug loading capacity			

### Polymer nanoparticles

Polymeric nanoparticles are substances with the sizes between 1 and 1000 nm, which include nanospheres, nanocapsules, and polymeric micelles (Woodman et al. 2021). Nanospheres have a core and an exterior composed of polymeric material, and the drug can be retained inside or adsorbed on the exterior surface; nanocapsules are a core–shell form with aqueous or oily cavities, and usually the drug is confined and surrounded by a polymer shell, which controls the release of the drug from the core at specific locations (Zielinska et al. 2020). Polymeric micelles have a core–shell structure composed of amphiphilic block copolymers which can be self-assembled in an aqueous phase environment. The core of polymeric micelles is hydrophobic, in which water-insoluble drugs can be harbored and show good drug stability. Some water-insoluble drugs had been successfully delivered with it (Majumder et al. 2020). The hydrophobic core can also be adjusted according to the optimal hydrophobic/lipophilic balance, size, stability of body circulation, drug-carrying capacity, etc. Compared to conventional drug delivery methods, nanosized micelles not only can escape renal rejection but also have better endothelial permeability. In addition, micelles can be more accurately localized to tumor tissue (Ghosh and Biswas 2021). In 2005, Abraxane entered the drug market as the first polymeric nanodrug. Similarly, two polymeric micelles named K911 and NK105, respectively, containing Adriamycin and Paclitaxel, were also introduced to the drug market (Aghebati-Maleki et al. 2020). β-Lapachone (β-lap) is a novel anticancer drug biologically activated by NAD(P)H:quinone oxidoreductase 1, which is specifically overexpressed in non-small cell lung cancer. Elvin Blanco et al. reported that the drug stability, bioavailability and targeted delivery of bioactivator β-lap can be achieved using biocompatible nanocarrier polymeric micelles. The small size of the β-lap micelles allows a good release of the drug in the tumor tissue. In mice bearing subcutaneous A549 lung tumors, the micelles-delivered β-lap concentrations were significantly prolonged in tumor tissue, resulting in delayed tumor growth and increased survival (Blanco et al. 2010). Sankha Bhattacharya’s study showed that polymeric nanoparticles conjugated with anti-EGFR monoclonal antibody and 5-fluorouracil (5-FU) had higher cellular uptake and higher cytotoxicity, and thereby have a good therapeutic effect on EGFR-positive colorectal cancer (Bhattacharya 2021). In addition, as a novel therapy polymeric nanoparticles was used to deliver low solubility and low bioavailability polyphenols for the treatment of skin cancers, such as melanoma (Vittala Murthy et al. 2022) (Table 2).

### Liposomes

Ordinary liposomes are bilayer spheres formed by self-assembly of amphiphilic molecules which heads are hydrophilic, and the tails are hydrophobic. In liposome the hydrophilic substances can be encapsulated in the hydrophilic core and the hydrophobic molecules can be encapsulated in the lipid bilayers. This amphiphilic property makes liposomes the choice for delivery of the drugs with different polarities (Large et al. 2021). In 1961, Bangham and Horne first described liposomes at the Babraham Institute in Cambridge, which opened the prelude to the study of liposomes (Sforzi et al. 2020). In fact, liposomes are the simplest biological cell mimics, and their main components include lipids and fatty acids, which are also present in cell membranes, so liposomes are considered to have good biocompatibility and biodegradability. Because of these properties, liposomes have been widely studied as carriers for drug delivery to tumor tissues. The pH value is one of the commonly used factors to stimulate liposomes. Acid-sensitive liposome components, such as fatty acylethanolamine (PE) or its derivatives, when exposed to an acidic environment, will lead to changes in the liposome structure, making the liposome structure change for drug release. The pH of tumor tissue is between 5.7 and 7.0, and the pH of normal human tissue is between 7.4 and 7.5, which provides a good opportunity for liposomes to release drugs in tumor tissue (Sang et al. 2021). Liposomes can be classified based on the size. Small unilamellar vesicles (SUVs) range in size from 20 to 100 nm; large unilamellar vesicles (LUVs) range in size from 100 to 1000 nm; Oligolamellar vesicles (OLVs) are between 100 and 1000 nm in size; multilamellar vesicles (MLVs) are larger than 500 nm in size; giant unilamellar vesicles (GUVs) are larger than 1000 nm in size; The multivesicular size of the bubbles can be from 1000 nm to several thousand nanometers (Sforzi et al. 2020). The use of liposome drug delivery systems in immunotherapy was divided into five categories by Zili Gu et al. First, it can be used for vaccine delivery to improve efficacy; second, it normalizes the tumor microenviroment by overcoming the tumor-driven immunosuppressive signals; Third, it modulates the tumor; fourth, it targets overexpressed surface molecules on tumor; fifth, it is combined with other treatments to form a combination therapy (Gu et al. 2020). It has been shown that the incorporation of up-conversion nanoparticles (UCNPs) and IR-1048 organic dye (NIR-II, 1000–1700 nm) into aptamer-modified lipid nanostructures (UCILA) can be used as computed tomography (CT) contrast agent to monitor the metabolic changes of lung cancer and to feedback the temperature of photothermal therapy (PTT) (Xu et al. 2021). As early as 1995, Doxil, a liposomal preparation of an anticancer drug doxorubicin, was approved by the FDA for the first time in the treatment of ovarian cancer and AIDS-related Kaposi’s sarcoma (Barenholz 2012). Over the years, increased liposome drug delivery systems have been used in clinical practice. Onivyde, a nanoliposomal formulation of Irinotecan, plays a huge role in malignancies, such as pancreatic, esophageal, gastric, and colorectal cancers, and was further recommended for patients with advanced solid tumors through clinical trials (Zhang 2016). Compared with traditional Irinotecan, Onivyde can better protect the drug, increase the concentration of the drug in tumor tissue and reduce the toxicity of host cells. Vyxeos® (CPX-351), a drug approved for the treatment of acute myeloid leukemia, is a liposomal form of Daunorubicin and Cytarabine, and clinical trial patients receiving Vyxeos® have shown a similar increase in survival. Compared with the conventional treatment group, the survival time was improved by about 4 months. The above three liposome-delivered drugs have been approved by the FDA for clinical use since 2015 (Almeida et al. 2020). In addition, Onpattro®, a recently approved liposomal therapy for the treatment of transthyretin-mediated (ATTR) amyloidosis, is a double-stranded siRNA formulated into lipid nanoparticles targeted to primary synthetic Hepatocytes at the site of TTR to inhibit the synthesis of TTR (Urits et al. 2020). The potential of polymeric lipid nanoparticles as drug delivery vehicles was further illustrated in the article by James C Kaczmarek et al. using polymeric lipid nanoparticles to deliver DNA or mRNA to lung endothelial cells (Kaczmarek et al. 2021). In one study, the use of podophyllotoxin-loaded lipid bilayer nanoparticles inhibited the production of death ligand 1 in lung cancer cells, not only reducing systemic toxicity, but also exhibiting immune and antitumor effects (Wang et al. 2022a, b). Liposome formulations of chemotherapeutic drugs are of interest, because liposomes can protect against drug toxicity and reduce the toxicity of encapsulated drugs to normal cells (Table 2).

### Gold nanoparticles

Consistent with the properties of gold, gold nanomaterials are also one of the most stable materials, and in the study of Mehak Jindal et al., gold nanoparticles therapy was noted to have the remarkable feature of the convenience of targeted drug delivery. Gold nanoparticles are very promising in cancer therapy and can be used as contrast agents, carriers of pharmaceutical products, radiosensitizers and photothermal agents, etc. (Jindal et al. 2020). The optical properties of gold and the quantum size of gold nanoparticles were reported by Michael Faraday as early as 1857. The size of gold nanomaterials ranges from 3 to 200 nm (Woodman et al. 2021). Metal or metal oxide nanoparticles are easily synthesized by physical, chemical or biological methods. Depending on the application of gold nanoparticles, they are made into various shapes and sizes, including gold nanospheres, nanocages, nanostars, nanoshells and nanorods, etc. Gold nanoparticles have high-sensitivity optical and electronic properties (Guo et al. 2017). Gold nanoparticles are considered as ideal radiosensitizers for radiotherapy due to high X-ray absorption and unique physicochemical properties. Multidisciplinary research was conducted to improve the design of gold nanoparticles to achieve better treatment effect (Chen et al. 2020). In the experiment of Romy Mueller et al., human lung cancer cell A549 was irradiated with gold nanoparticles, and by monitoring the expression of carcinoembryonic antigen (CEA) on the surface of lung cancer cells, it was found that the expression of CEA increased with the increase of radiation dose and time. This experiment shows that gold nanoparticles can significantly increase the expression of CEA (Mueller et al. 2020). Zhidong Teng et al. selected gold nanoparticles as an adjuvant in virus-like particle (VLP) vaccine because of the minimal toxicity of gold nanoparticles in vivo and in vitro, and the enhanced immune response (Teng et al. 2021). Functionalized gold nanoparticles can be used as biosensors to detect the content of human insulin. During the outbreak of the novel coronavirus, nanoparticles can also be used as nanoprobes to detect the nucleic acid (Fan et al. 2020). In 2004, the use of colloidal gold nanoparticles for targeted drug delivery to tumor tissue was first described by Giulio F Paciotti et al. This drug delivery system consists of thiol-derived PEG (PT) molecules, gold nanoparticles and recombinant human TNF (tumor necrosis factor), abbreviated as PT-cAu-TNF. After intravenous injection, PT-cAu-TNF rapidly accumulated in animal MC-38 colon cancer tumors, while in other normal tissues, such as liver and spleen, there was almost no aggregation. Compared with TNF alone, PT-cAu-TNF showed reduced toxicity to normal tissues and the enhanced antitumor effect at low doses (Paciotti et al. 2004). In the article by Jacob D. Gibson et al., they first described the synthesis of the drug functionalized nanoparticles by covalent attachment of the chemotherapeutic drug Paclitaxel to the surface of 2 nm gold nanoparticles (Gibson et al. 2007). Colloidal gold has been reported to treat rheumatoid arthritis, and radioactive gold nanoparticles were reported to treat liver cancer (Kumar et al. 2013). Gold nanoparticles have a great role in the treatment and diagnosis of various cancers, including but not limited to lung cancer, breast cancer, liver cancer, cervical cancer, rectal cancer, etc. (Liu et al. 2021). Related studies have also demonstrated that GNS@CaCO3/Ce6-NK has a significant targeting effect and superior therapeutic effect on lung cancer A549 tumor-bearing mice (Liu et al. 2019) (Table 2).

### Quantum dots

Quantum dots (QDs) are nanoscale semiconductor crystals, which are alloyed nanocrystal colloids or core shells composed of II–VI or III–V elements in the periodic table. QDs were first discovered by Russian physicist Alexei in the 1980s. After that, QDs have been gradually developed in different medical fields, such as biomarkers, medical imaging, disease detection, cancer treatment, drug delivery, etc. There are many types of QDs, such as carbon QDs, graphene QDs, silicon QDs, gold QDs, etc. (Kargozar et al. 2020). QDs have many characteristics that other nanoparticles do not have. First, their sizes, ranging from 4 to 12 nm, are smaller than most nanoparticles. The smaller QDs make them more permeable through tight junction, such as blood–brain barrier (Gour et al. 2021). Second, QDs exhibit special photostability, photoluminescent and fluorescent activity due to their unique photophysical properties (Gour et al. 2021). In 1998, QDs were first applied to the fluorescent labeling of biological species (Wang and Chen 2011; Xu et al. 2021). Finally, QDs has larger specific surface area for more drugs loading (Henna and Pramod 2020). QDs have been used in nanomedicine. Doxorubicin (DOX) and methotrexate (MTX) are commonly used drugs for the treatment of cancer. Due to their poor solubility, they cannot be delivered to the desired tissues in free form. When combined with graphene QDs, they were well-entered into the desired tissues (Chung et al. 2021). In 2006, Noriyoshi Manabe conjugated QDs with the antihypertensive drug Captopril (QD-cap) to study the effect of QD-cap in vivo and in vitro. With QDs fluorescence properties the kinetics and dynamics of QD-cap were analyzed in blood and organs (Manabe et al. 2006). In addition, the peptides conjugated to QDs resulted in their selective accumulation in tumor tissues and blood vessels. Coupling of QDs with monoclonal antibodies against prostate-specific membrane antigens was used to detect the prostate cancer in mice. In addition, coupling of QDs with anti-fetoprotein antibodies was used to diagnose liver cancer (Pleskova et al. 2018). The above studies can all prove that QDs can increase the therapeutic effect of anticancer drugs, proving the possibility of quantum dot drug delivery system (Table 2).

### Dendrimers

Dendrimers were first discovered by Donald Tomalia in 1985. They are composed of three parts: the core, the inner layer of repeating molecular units called dendrites, and the surface end groups. Because of this special structure, it has diverse ways of carrying drugs. Drugs can be loaded into the inner core or attached to the surface for delivery to the tissue (Dias et al. 2020). The size of dendrimer is between 2 nm and 10 nm. Dendrimer is used in nanomedicine in two types. One is a low molecular weight form, which is composed of a single dispersed symmetrical branch, and the other is the high molecular weight form, which is composed of dendrimers, hyperbranched and brush polymers (Woodman et al. 2021). The atom or group of atoms in the middle of a dendrimer surrounded by symmetrical branches is called dendrites, which grow from the inside out. The unique properties of dendrimers make them used in different biomedical fields, such as diagnostic reagents, delivery of drugs and proteins, and the development of vaccines, etc. In particular, the three-dimensional structure of dendrimer can combine multiple drugs to form active complexes, which are more effective than free drugs alone (Mittal et al. 2021). There are many functional groups on the surface of dendrimers, and the inner cavity also has a high loading capacity for drugs, which can improve the availability of drugs through covalent or non-covalent connection. Some dendrimers also have their own medicinal value and can be used for antifungal, antibacterial, etc. (Chis et al. 2020). The dendrimers were found to be toxic to some cell lines and the dendrimers with higher generation and increased positive surface charges were more toxic. Interestingly, the toxicity can be significantly reduced by different chemical modifications on its periphery, such as polyethylene glycol (PEG), acetyl group, carbohydrates, etc., while the activity of the dendrimer is not affected. By modification, the dendrimers can be toxic to cancer cells without affecting normal cells. Therefore, dendrimers have great potential for drug delivery (Janaszewska et al. 2019). In 2007, Vivagel® as the first dendrimer nanoparticle was introduced to the pharmaceutical market. The drug combined with the dendrimer prevents the virus from attaching to host cells for the prevention of human immunodeficiency virus and herpes virus spread (Aghebati-Maleki et al. 2020). Dendrimers are often combined with various antitumor drug molecules. The conjugation of doxorubicin (DOX) with dendrimers through acylhydrazone bonds can improve lung tumorigenesis. The conjugation of paclitaxel (PTX) with dendrimers increases specificity and cytotoxicity in breast cancer. The active cisplatin molecules coupled with dendrimers can be specifically targeted to ovarian tumor cells. Angiotensin conjugated with dendrimers forms stable complexes to inhibit cardiovascular diseases, etc. (Chis et al. 2020; Sandoval-Yanez and Castro Rodriguez 2020). In conclusion, in the drug delivery system, dendrimers not only have high drug-loading capacity but also can improve the selection of biological targets and specificity to tumor tissue (Table 2).

### Carbon nanotubes

In 1976, Oberlin et al. first reported the composition of carbon nanotubes. Until 1991, the interest on it was aroused by the research by Iijima et al. and further promotes the development of the related nanotechnology (Saito et al. 2009). There are two types of carbon nanotubes. One is single-walled carbon nanotube (SWCNT), which is a tubular structure made of a layer of graphene rolled into a cylinder. Its diameter ranges from 0.4 nm to 40 nm. Because of good stability and flexibility it was paid much attention on. The other is multi-walled carbon nanotubes (MWCNTs), which are composed of multiple sheets forming concentric cylinders with diameters ranging from 2 to 100 nm (Negri et al. 2020). Carbon nanoparticles have the characteristics, such as high surface area, high loading capacity, stability, easy functionalization, cell membrane penetration and good cellular uptake, which makes it one of the preferred carriers for drug delivery systems (Chadar et al. 2021). Carbon nanoparticles suspension drip combined with parathyroid vascular preservation can effectively reduce the injury of parathyroid gland after thyroidectomy (Yin et al. 2020). Small molecules can be easily functionalized on or inside the carbon nanoparticle walls. Some studies showed that drugs delivered through carbon nanoparticles can be more efficiently absorbed by cells and trigger effective antifungal activity. To achieve a more significant effect in cancer treatment, carbon nanotubes can be easily combined with different bioactive molecules, functionalized, by covalent bond or non-covalent supramolecular assemblies for targeting (Chadar et al. 2021). Carbon nanotubes are of great significance in the treatment of diseases, such as malaria, Alzheimer’s disease, infectious diseases and asthma, and increased responsiveness in cardiovascular and pulmonary disease (Khan et al. 2021). In 2004, Shi Kam et al. first showed that carbon nanoparticles can be derivatized and be adhered to small molecules and proteins. Then, in 2005, Venkatesan et al. demonstrated that the bioavailability of EPO in rats can be improved by carbon nanoparticles as a drug delivery system. There was also a report that DNA plasmids was immobilized on carbon nanoparticles and introduced into target cells, which provided strong support for gene transfer (Saito et al. 2009). It has been reported that a drug delivery system of CNT:1,2-distearoylphosphatidylethanolamine-methyl PEG conjugates can not only release the drug in tumor tissue but also reduce the toxicity of gemcitabine hydrochloride (Deshmukh et al. 2020). The cardiotoxic anticancer agent Doxorubicin (Dox), an anthracycline anticancer drug, can be easily adsorbed on the surface of carbon nanotubes through π–π stacking interactions, and loaded onto the modified CNTs structure, which can not only adjust the dose but also increase its uptake by tumor cells, thereby reducing toxicity and achieving high efficiency (Chadar et al. 2021) (Table 2).

The formation of tumors is promoted by numerous factors, such as physical, chemical, biological, genetic, etc. This process is closely related to the structure and function of the tumor microenvironment (TME), which includes blood vessels, immune cells, and fibroblasts, signaling molecules and extracellular matrix, etc. The traditional therapeutic effect is always limited by the limited retention time in the TME (Kim et al. 2022). Xiangzhou Meng et al. proposed a nano-delivery system based on lung cancer cell membrane to release relevant substances into the tumor microenvironment (Meng et al. 2021). In the article, nanoparticles were used to enhance the stability of the drug, and to target the drug to the tumor, which can make the drug more permeable in the tumor tissue, elongate the residence time in the tumor tissue, and make the immune cells and monoclonal antibodies better circulating in the TME. The antigenic properties of nanoparticles enhanced tumor immune responses (Rodallec et al. 2018; Yang et al. 2021). T cells are a vital component of the tumor microenvironment. A novel T cell membrane-coated nanoparticle (TCMNP) was applied in lung cancer immunotherapy (Kang et al. 2020). One article reported two bioreactive nanoparticles and CD40 agonists synergistically promoted antitumor T cell responses and reversed the immunosuppressive tumor microenvironment (Ling et al. 2021). Macrophages are also a vital component in tumor microenvironment. Yonghui Wang et al. used M1-like macrophages (NP@M1) containing celastrol nanoparticles to successfully deliver celastrol drugs, and the nanoparticles also showed the killing effect on tumor cells (Wang et al. 2022a, b). Another biohybrid RNAi peptide nanoparticle was reported efficiently and selectively to induce tumor-associated macrophages to exert tumor suppressive effects (Conde et al. 2015). Hend Mohamed Abdel-Bar et al. also used a nano-delivery system to delay tumor growth in a mouse lung tumor model (Abdel-Bar et al. 2021). A new drug delivery system p28-NPs-GEF can selectively reduce A549 cells activity. It provides the possibility to improve the treatment efficacy of lung cancer (Garizo et al. 2021). In the review by Jenna C Harris et al., it was also proposed that nanoparticles have excellent targeting ability. Compared with the drug delivery without nanoparticles, the nanoparticles encapsulated drug delivery reduced the amount of drugs in normal tissues, and increased the drug concentration in tumor microenvironment (Harris et al. 2019). By co-assembly of cisplatin (CDDP) and metformin (MET) to form nanoparticles, The nanoparticles formed by co-assembly of cisplatin (CDDP) and metformin (MET) exhibited high aggregation in tumor tissue in Lewis lung cancer mice to inhibit tumor growth and improve mouse survival (Yang et al. 2020).

Nanomedicine is a biomimetic nanotechnology that involves various disciplines, such as biology, medicine, and chemistry. It has significant advantages in targeting drug delivery by comparison with other drug delivery modalities. Nanoparticles have a smaller size compared to conventional biomolecules, which will facilitate the nanoparticle to react with biomolecules on the cell surface or within cells. Because of its biological safety, drug loading and physical properties most of the nanoparticle-mediated therapies are multifunctional, with few adverse reactions and good curative effects (Carrasco-Esteban et al. 2021; Doroudian et al. 2021) Table 2.

## Application of nanoparticles in the treatment of lung cancer

**Fig. 1:**
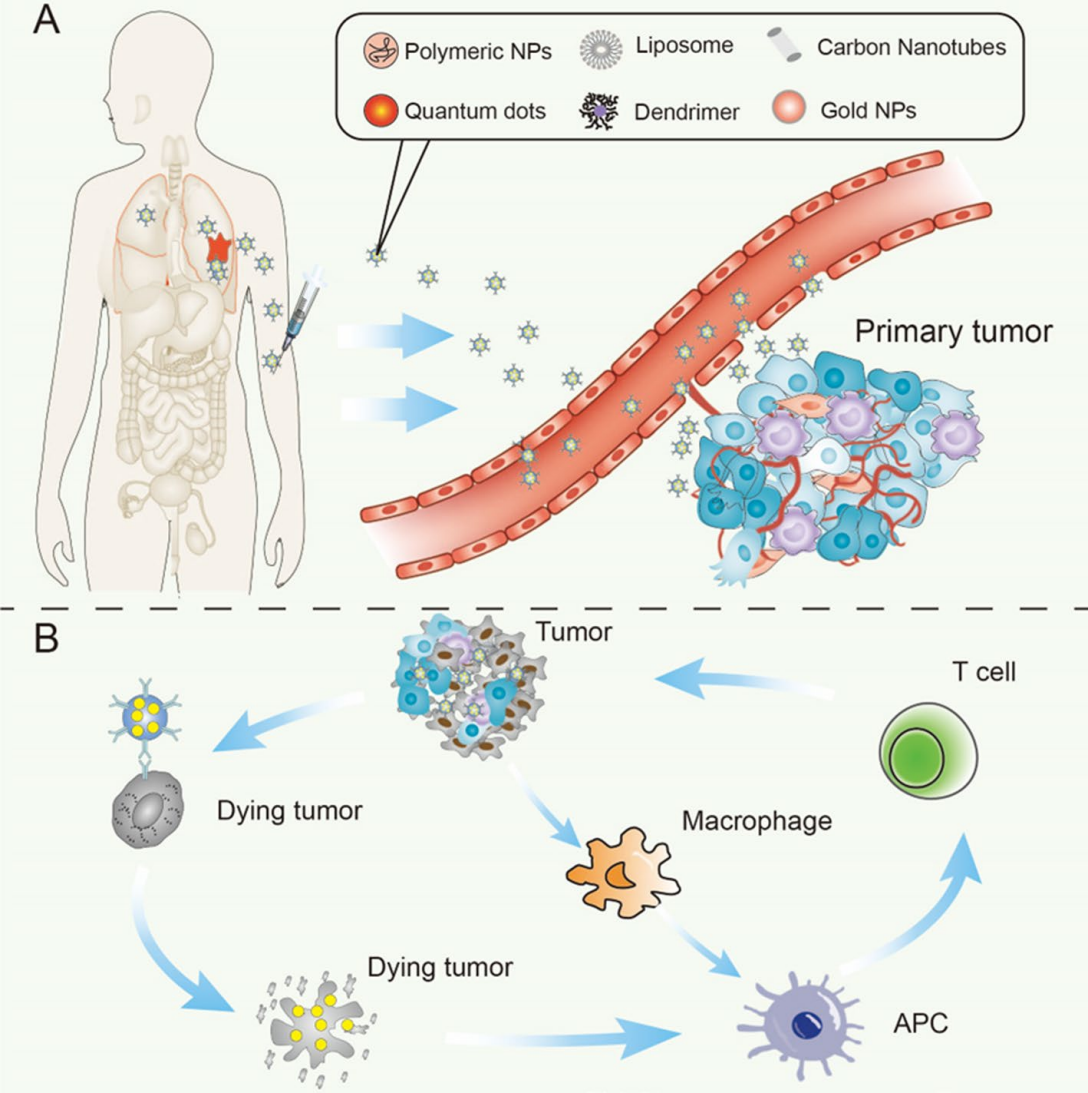
Common types of nanoparticles and their pathways through the body (**A**) and mechanisms of action of nanoparticles in tumor tissue (B)

The drug-conjugated nanoparticles can passively or actively target the tumor tissue. In passive targeting, nanoparticles remain in tumor tissue and enhance permeability, while reducing lymphoid tissue clearance. In active targeting, ligands on the surface of nanoparticles specifically bind to receptors on the tumor surface, accurately increasing drug uptake in tumor tissues and reducing toxicity to normal tissues (Doroudian et al. 2021). When the drugs are delivered to the tumor tissue, the tumor cells rupture and then trigger a series of immune responses. The tumor-associated antigens released from the broken tumor tissue are processed and presented by the antigen-presenting cells (APCs), which are then delivered to T cells to produce an immune response. Of course, original tumor tissue can also transmit signals to APC for immune response (Li et al. 2021) (Fig. 1). Nanoparticles have shown remarkable effects in cancer treatment as a good carrier to safely deliver drugs to tumor tissues to achieve immune-targeted therapy. In recent years, nanoparticles have also been used in the treatment of lung cancer. A series of clinical trials and preclinical studies of the nanoparticles-conjugated drugs have shown promising results. These nanoparticle-based drugs are directly or indirectly involved in the treatment of lung cancer.

### Preclinical studies

Nanomedicine has many advantages in targeted delivery and treatment of lung cancer, and a series of animal experiments have been carried out to demonstrate the role of nanoparticles in the treatment of lung cancers (Table 3). Gregory E Holt et al. proposed that the ability of NSCLC-NP to selectively transfect lung cancer can be used as a starting point for targeted therapy of lung cancers (Holt and Daftarian 2018). Small cell lung cancer is an aggressive lung cancer of neuroendocrine origin which is closely related to smoking. The first-line chemotherapies have a certain role in the treatment, but it is prone to recurrence and drug resistance over time because of the chemoresistant cell clones (Bernhardt and Jalal 2016). Ryosuke Tanino et al. found that ZnO nanoparticles are toxic to small cell lung cancer, can reduce tumor cell viability in mice, while there were no obvious side effects during the trials, making ZnO nanoparticles a potential drug for the treatment of small cell lung cancer (Tanino et al. 2020). Liposomes and polymer nanoparticles are widely used in drug delivery due to their unique physical properties. In the article by Lei Wei et al. Doxorubicin and infrared dye were encapsulated by PD-L1 antibody conjugated liposome–polymer nanoparticles (called PD-L1 antibody nanoparticles). Animal experiments with PD-L1 antibody nanoparticles were carried out on nude mice model. By the small animal imaging methods it showed that PD-L1 antibody nanoparticles were efficiently targeted on tumor cells. Similarly, PD-L1 antibody nanoparticles was also targeted to A549 cells. The study also showed that PD-L1 antibody nanoparticles killed the tumor cells and inhibited tumor growth effectively. This study demonstrated that PD-L1 antibody nanoparticles are a superior drug delivery vehicle (Wei et al. 2021). As mentioned above, tumor microenvironment is different from the normal tissue environment. The nanoparticles that respond to the stimuli of the tumor microenvironment have been widely studied in the treatment of tumors. Roshni Iyer et al. developed Cisplatin-loaded glutathione (GSH)-responsive polyurethane nanoparticles (Cisplatin-GPUs). The Cisplatin can be released once the Cisplatin-GPUs arrived at the tumor microenvironment with high GSH level. With the xenograft A549 lung tumor mice model the study showed that Cisplatin-GPUs significantly inhibited the tumor growth by comparison with free Cisplatin (Iyer et al. 2020). Xueling Guo et al. developed a multifunctional chitosan nanoparticle in which methotrexate can be encapsulated through hydrophobic interactions. By intravenous injection of such nanomedicine into BALB/c mice, the methotrexate was significantly accumulated at tumor sites and inhibited lung tumor growth (Guo et al. 2018). These studies provide a support for targeted drug delivery of nanoparticles to inhibit tumor cells for therapeutic purposes. Hyperthermia is also a method of treating tumors. One study showed that hyperthermia can be induced by exposure of iron oxide nanoparticles in targeted pulsed electromagnetic fields (PEMF) for the treatment of lung cancer on xenografted mouse model (Baskar et al. 2017). In the research by Tian Zhong et al., sorafenib (SORA) and crizotinib (CRIZ) were encapsulated with the polymer NPs (referred to as SORA-CRIZ@NPs) to treat the lung cancer in Nude mouse xenograft model. The results showed SORA-CRIZ@NPs nano-delivery system can inhibit the development of tumor, elongate the survival period, and minimize toxic side effects (Zhong et al. 2021). Kiran Jyoti et al. studied the effect of MUC-1 peptide-loaded poly(lactide-co-glycolide) nanoparticles (MUC-1 peptide-PLGA-NA-NPs) on NSCLC. The results showed that uptake of MUC-1 peptide-PLGA-NA-NPs was enhanced in murine macrophages and demonstrated the antitumor potential of MUC-1 peptide-PLGA-NA-NPs by the inhalation route of administration in NSCLC xenograft models (Jyoti et al. 2020). In another study antitumor effect of the lipid nanoparticles loaded with lumefantrine and calcium phosphate nanoparticle (LF-CaP-Ls) was examined in a mouse model. The results showed that LF-CaP-Ls has a high efficacy in reducing tumor progression in the mice (Sethuraman et al. 2021). To improve the therapeutic effect of etoposide and cisplatin in NSCLC, Maofan Zhang et al. developed a polymer nanoparticle formulation of cisplatin and etoposide for delivery into a mouse model. It showed more effective results than free drug and the reduced drug toxicity (Zhang et al. 2021). Jillian L Perry et al. used PRINT (particle replication in nonwetting templates) nanoparticles as carriers to deliver CpG via orotracheal instillation into the lungs of the two murine orthotopic metastasis models. The results show that this method prolonged the retention time of CpG in the lungs, improved the anti-cancer efficacy, and had incidental therapeutic effects on systemic diseases (Perry et al. 2020). In the study by Neha N Parayath et al., miR-125b conjugated hyaluronic acid-poly (ethyleneimine) (HA-PEI)-based nanoparticles showed the ability to reprogram TAMs (tumor-associated macrophages) into an antitumor/pro-inflammatory (M1) phenotype in a genetically engineered NSCLC mouse model (Parayath et al. 2018). Although chemoradiotherapy is an indispensable treatment option in tumor treatment, due to distinct reasons, such as high toxicity and drug resistance, the proportion of chemoradiotherapy treatment has decreased. The above applications of chemoradiotherapy combined with nanotechnology showed the potential in lung cancer treatment.

**Table 3:** Preclinical studies of nanoparticles in the treatment of lung cancer

Drugs	Tumor Type	Drug delivery method	Test site	Models	Test results	References
ZnO nanoparticles	SCLC	i.v	In vivo	Orthotopic mouse model	Toxic to mouse lung cancer cells without any side effects after the trial	(Tanino et al. 2020)
PD-L1 antibody nanoparticles	A549	Small animal imaging method	In vivo	Nude mouse model	Good targeting to A549 tumor cells and easy to be absorbed	(Wei et al. 2021)
Glutathione-responsive polyurethane nanoparticles	A549	i.v	In vivo	Xenograft mouse model	Shows growth inhibitory effect on lung tumors	(Iyer et al. 2020)
Multifunctional Chitosan Poly Nanoparticles	A549	i.v	In vivo	BALB/c mouse model	Methotrexate accumulates at tumor sites and significantly inhibits tumor growth	(Guo et al. 2018)
Magnetic Nanoparticles	A549		In vitro	Xenograft mouse model	Nanoparticles are cytotoxic and enhance tumor cell uptake	(Baskar et al. 2017)
SORA-CRIZ@NPs	A549	i.v	In vivo	Xenograft mouse model	Significantly reduces tumor survival with minimal side effects	(Zhong et al. 2021)
MUC-1 Peptide-PLGA-NA-NPs	A549		In vitro	Xenograft mouse model	Enhanced cellular uptake	(Jyoti et al. 2020)
LF-CaP-Ls	Lung cancer	i.v	In vivo	Mouse model	Effective on mouse tumors, reduce tumor progression, and have basic anti-cancer effects	(Sethuraman et al. 2021)
Etoposide and Cisplatin Dual Drug-loaded Nanoparticles	NSCLC		In vivo	Mouse model	More effective and less toxic than free drug	(Zhang et al. 2021)
Nanoparticle-bound CpG	NSCLC-344SQ KAL-LN2E1	Oral tracheal drip	In vivo	Mouse Orthotopic Metastasis Model	Extend the retention time of CpG in the lung, improve the anti-tumor factor of the lung, and have incidental therapeutic effect on systemic diseases	(Perry et al. 2020)
HA-PEI	NSCLC	i.p	In vivo	Genetically engineered mouse models	HA-PEI successfully transfected mouse and mouse lung TAM	(Parayath et al. 2018)

### Clinical application

The following section will introduce the clinical application of nanoparticles in the treatment of lung cancer (Table 4). Paclitaxel is a classic drug for the treatment of tumors, it is widely used in lung cancer, breast cancer, esophageal cancer and other diseases, However, due to the hydrophobicity of paclitaxel, it needs to be administered in combination with Cremphor EL (CrEL) and an ethanol carrier to enhance drug solubility (Petrelli et al. 2010). However, the use of Cremphor EL has caused a series of adverse reactions (Stinchcombe 2007). To avoid the occurrence of these adverse reactions, new paclitaxel delivery systems were developed, such as paclitaxel albumin nanoparticle formulation (nab-paclitaxel), which was developed more than a decade ago as a CrEL-free formulation, which showed better performance, high reactivity and tolerance in NSCLC patients (Yardley 2013). In Charity D Scripture et al., it is known to be less toxic than Cremophor's paclitaxel in mice and exhibits linear pharmacokinetics at doses of 135–300 mg/m^2^ (Scripture et al. 2005). It is approved in the United States for the treatment of three solid tumors, metastatic breast cancer, locally advanced or metastatic non-small cell lung cancer, and metastatic pancreatic cancer (Kundranda and Niu 2015). In a phase III clinical trial (NCT01620190), 503 patients with advanced, previously treated NSCLC were randomized 1:1 to 252 patients, in the nab-paclitaxel arm on days 1, 8 and 15 nab-paclitaxel (100 mg/m^2^), 251 patients in the docetaxel group received docetaxel (60 mg/m^2^) on the first day, a 21-day cycle. Through a monitoring observation study of nearly 3 years, It can be known that the median OS of the nab-paclitaxel group was 16.2 months (95% CI 14.4–19.0) and the docetaxel group was 3.4 months (95% CI 2.9–4.1), and in some serious adverse reactions such as Febrile neutropenia was reported in 2% of the nab-paclitaxel group and 22% of the docetaxel group, while peripheral sensory neuropathy was reported in 10% of the former and 1% of the latter. From these data, the OS data of nab-paclitaxel are not lower than the docetaxel group, so nab-paclitaxel can be used as a treatment option (Yoneshima et al. 2021). In a phase II clinical trial of NCT01620190, to determine the response rate of NAB-paclitaxel in patients with advanced NSCLC with EGFR mutation after first-line treatment with IGFR tyrosine kinase inhibitor (TKI), nab-paclitaxel was administered intravenously over 30 min on day 1, day 8, and day 15 to 26 patients, 28 days as a course of treatment. at up to 60 weeks of evaluation, serious adverse reactions such as vascular disease were found in 3.85% (1/26), of course, other adverse reactions also existed, including decreased neutrophil count, leukopenia, peripheral neuropathy, etc. The combination of carboplatin and paclitaxel has the effects of high activity, convenience of administration, etc., which can interact in pharmacodynamics and pharmacokinetics, and has the effect of preserving platelets (Calvert 1997). In a phase II clinical trial of NCT00553462, to investigate the response of patients with unresectable stage III non-small cell lung cancer to carboplatin and nab-paclitaxel, as well as radiation therapy and erlotinib, 78 participants Intravenous Paclitaxel on days 1 and 8, carboplatin intravenously on day 1, 21 days as a course,for two courses, Erlotinib hydrochloride orally once daily on day 43, and weekly After 5 days of concurrent radiotherapy, the results showed that 25.33% (19/75) of serious adverse reactions occurred after 2 years of follow-up, including heart disease, blood and lymphatic system diseases, and gastrointestinal diseases. Similarly, the NCT00729612 and NCT00544648 clinical trials investigated the treatment of lung cancer with nab-paclitaxel, carboplatin, and radiotherapy. The phase II clinical trial of NCT00729612 was for patients with stage IIIB, stage IV or recurrent non-small cell lung cancer. The first day Inject nab-paclitaxel and carboplatin, respectively, 21 days for a course of treatment, through follow-up, the proportion of serious adverse reactions was 3.17% (2/63). Phase I and Phase II clinical trials of NCT00544648 for patients with unresectable stage III non-small cell lung cancer treated nab-paclitaxel in combination with carboplatin and radiotherapy. The follow-up showed that the proportion of serious adverse reactions in stage I was 50% (5/10), 50% (1/2) in stage II. Lung cancer is a global problem. In the past, the incidence rate in North America was always at the top, and it was the second most diagnosed cancer in the United States (Hoy et al. 2019). Mortality has been ranked first among malignant tumors (Oncology Society of Chinese Medical and Chinese Medical Association Publishing 2021). Of course, the occurrence of lung cancer is related to the development of the country and the social economy. Therefore, nab-paclitaxel was used in the two phase III clinical trials of NCT02775435 and NCT03875092. Co-treatment of adult NSCLC with carboplatin with or without pembrolizumab, the former 599 participants, all from North America, were injected with pembrolizumab 200 mg before chemotherapy, followed by intravenous injection of nab-paclitaxel and carboplatin on the first day, 21 days is a cycle, the proportion of serious adverse reactions in the study results was 40.65% (113/278) and the proportion of serious adverse reactions in the placebo control group was 38.21% (107/280). In the NCT03875092 trial, 125 participants were all Asian, and their drug injections were the same as the former. In the study results, the proportion of serious adverse reactions in the experimental group was 52.31% (34/65), and the proportion in the placebo control group was 36.67% (22/60). Pemetrexed is a new type of multi-targeted antifolate drug, which has certain activity when it is used in SCLC (Socinski 2005). Strong activity has also been shown in solid tumors, such as cervical cancer, colorectal cancer, head and neck cancer, and bladder cancer (Adjei 2003). In a Phase I Phase II clinical trial of NCT00470548, nab-paclitaxel was combined with Pemetrexed to observe their effect in treatment-stage non-small cell lung cancer. Intravenous 10-min Pemetrexed and 30-min nab-paclitaxel, respectively, in a 21-day cycle, in the second phase, received the same dose within the MTD range as the first phase, and the results of the study showed that phase I adverse events The incidence of adverse events was 8.33% (1/12), and the incidence of stage II adverse events was 16.22% (6/37). Another nanomedicine for the treatment of advanced NSCLC is CRLX101, which is a nanoparticle of camptothecin (CPT) conjugated to a cyclodextrin-based polymer. CPT is not used in cytotoxicity due to its unfavorable physicochemical effects. It was used in anti-tumor in the early stage, but when it was made into a nano-formulation, more CPT was exposed in tumor tissue and the side effects were minimized (Gaur et al. 2014; Schmidt et al. 2020). CRLX101 is widely used, and has certain efficacy in advanced renal cancer, gastric cancer, ovarian cancer, and triple-negative breast cancer, and some clinical studies have been carried out. In Thomas Schluep et al. review, three weekly doses of CRLX101 had a pronounced antitumor effect, tumor regression was observed in all H1299 tumor-bearing animals (Schluep et al. 2006).The activity and safety of CRLX101 in patients were evaluated in a randomized phase II trial, NCT01380769. The incidence of serious adverse reactions was 12.37% (12/97) in 157 participants who received 15 mg/m2 intravenously every other week after 18 months of follow-up. Neuroendocrine tumors include typical carcinoid (TC), atypical carcinoid (AC), large cell neuroendocrine carcinoma (LCNEC), and small cell carcinoma (SCLC) (Metovic et al. 2021), with the lung being the second most common after the gastrointestinal tract Primary site of neuroendocrine tumors (Melosky 2018). ABI-009 (protein-bound rapamycin nanoparticles) is an unapproved U.S. Food and Drug Administration (FDA) for the treatment of advanced malignant neuroendocrine tumors of the lung, gastrointestinal tract and/or pancreas. The NCT03670030 phase II clinical trial investigated the safety of ABI-009. Five participants were given ABI-009 intravenously on the first and eighth days, with a 21-day cycle. The results of the study indicated that the probability of serious adverse reactions was 60.00% (3/5), including nervous system diseases, gastrointestinal diseases and so on.

**Table 4:** Clinical application of nanoparticles in the treatment of lung cancer

Nanoparticle-Based Drugs	Other drugs	Allocation	Way of administration	Serious adverse reactions	Stage	State	Numbering
Nab-paclitaxel	TKI	Not applicable	Intravenous injection of nab-paclitaxel over 30 min on d1, d8, and d15	3.85% (1/26)	Phase II	Completed	NCT01620190
Nab-paclitaxel	Carboplatin; Erlotinib Hydrochloride	Not applicable	Intravenous injection of nab-paclitaxel for 30 min on day 1 d1 intravenous carboplatin 30 min Oral erlotinib hydrochloride after d43, once daily	25.33% (19/75)	Phase II	Completed	NCT00553462
Nab-paclitaxel	Carboplatin	Not applicable	On day 1, intravenous injection of nab-paclitaxel for 30 min, carboplatin for 1–2 h	3.17% (2/63)	Phase II	Completed	NCT00729612
Nab-paclitaxel	Carboplatin	Not applicable	d1, d8, d15, d22, d29, d36, d43 intravenous injection of nab-paclitaxel and carboplatin, respectively	I: 50% (5/10) II: 50% (1/2)	Phase I II	Termination	NCT00544648
Nab-paclitaxel	Carboplatin; Pembrolizumab	Random	Intravenous pembrolizumab before chemotherapy, nab-paclitaxel and carboplatin intravenously on day 1	40.65% (113/278)	Phase III	Active, not recruiting	NCT02775435
Nab-paclitaxel	Carboplatin; Pembrolizumab	Random	Intravenous pembrolizumab before chemotherapy, nab-paclitaxel and carboplatin intravenously on day 1	52.31% (34/65)	Phase III	Active, not recruiting	NCT03875092
Nab-paclitaxel	Pemetrexed	Non-random	I: Intravenous injection of nab-paclitaxel for more than 30 min, pemetrexed for more than 10 min II: Receive the same phase I drugs within the MTD range	I: 8.33% (1/12) II: 16.22% (6/37)	Phase I II	Termination	NCT00470548
CRLX101		Random	CRLX101 was administered intravenously at 15 mg/m^2^ every other week	12.37% (12/97)	Phase II	Completed	NCT01380769
ABI-009		Not applicable	Intravenous injection of ABI-009 on d1 and d8	60.00% (3/5)	Phase II	Termination	NCT03670030

Further improving the prognosis of lung cancer patients through nanoparticle drug delivery systems. This novel therapeutic modality may play a key role in more targeted and personalized medicine in the future. However, there will be many difficulties in this process. For example, in the development of multifunctional nanotherapeutic drugs to improve cancer nanomedicin clinical trials, industrial large-scale production of NPs has become more challenging as NPS become more complex in terms of structure and formulation. Moreover, due to the complexity of these nano-formulations, it is more difficult to control their physicochemical properties, and the effects of different materials on the human body are also unknown, we cannot be sure that there are no toxic side effects during the use. Therefore, the rational design of nanomaterials can optimize the physicochemical properties of NPs in terms of improving the absorption capacity, reducing cell efflux, and reducing toxicity, so as to improve the therapeutic effect. In addition, the effects of NPs on the immune system, drug stability issues, and scalability difficulties are also difficult to predict, so each stage of the nanomedicin manufacturing process needs to be carefully monitored and improved to ensure reproducible, stable, and efficient synthesis. This calls for a better understanding of the biological processes and advances in nanotechnology, as well as the development of more clinically relevant animal models to optimize the properties and delivery regimens of nanomedicine and to develop more biocompatible and effective nanomedicine drugs.

## Summary and outlook

The occurrence of lung cancer affects people all over the world. Lung cancer is one of the leading causes of death despite ongoing treatments. Effective treatment of lung cancer has become an urgent problem that needs to be solved. In addition to conventional treatments, such as surgery, radiotherapy and chemotherapy, Nano research for the treatment of lung cancer is still developing rapidly, especially the use of nano-drug delivery systems to improve the stability of drugs and successfully target drugs to tumor tissues, due to the adjustability, enhancement and retention effects of nanoparticles, the treatment effect is greatly improved, and the damage to the surrounding tissue is reduced. Nanoparticles such as polymers, liposomes, QDs, dendrimers, gold nanoparticles, etc. have now been studied, not only in lung cancer, but in a variety of tumors to accurately deliver drugs with minimal side effects. A series of preclinical studies have combined conventional drugs for the treatment of lung cancer with nanoparticles and used this drug delivery method to solve problems, such as drug solubility and toxicity. Compared with the use of free drugs alone, it can more effectively inhibit the growth of tumors and significantly improve the survival period of experimental animals. Paclitaxel is a commonly used anti-tumor drug. In clinical trials based on nanoparticles for the treatment of lung cancer, most of them combine paclitaxel with nanoparticles to target lung cancer. It has unique advantages in anti-lung cancer. Based on these characteristics of nanoparticles, their status in the medical field is gradually rising. To make nanoparticles more widely used in medicine, it is necessary to pay more attention to the use of materials that make up nanoparticles, and to control the toxicity of nanomaterials to achieve no harm to the human body, rational design of nanomaterials to increase cellular uptake, reduce cellular efflux, and achieve biocompatibility, etc. Solving these problems requires cooperation between bioengineering and biomedicine and even more disciplines. The development of better nanoparticles will bring a new dawn to the treatment of lung cancer.

## Data Availability

Not applicable.
